# A Systematic Review and Network Meta-Analysis of the Outcomes of Patients With Total Knee Arthroplasty Using Cemented, Uncemented, or Hybrid Techniques

**DOI:** 10.7759/cureus.47299

**Published:** 2023-10-18

**Authors:** Zien Alabdin Fozo, Ahmed Hussein Ghazal, Ibrahim Kamal, Mona Muhe Eldeen Eshag, Mahmoud M Elhady, Mohamed Hesham Gamal, Khalid Mohamed fisal, Khaled Mohamed Ragab

**Affiliations:** 1 Orthopaedics, Ysbyty Gwynedd Hospital, Bangor, GBR; 2 Orthopaedics, Northwick Park Hospital, London North West University Healthcare NHS Trust, London, GBR; 3 General Medicine, Al-Azhar University, Alexandria, EGY; 4 General Practice, Faculty of Medicine, University of Bahri, Khartoum, SDN; 5 Orthopaedics, Faculty of Medicine, Benha University, Qalubiya, EGY; 6 Pharmacology and Therapeutics, Faculty of Pharmacy, Tanta University, Elgharbia, EGY; 7 Pharmacology and Therapeutics, Faculty of Pharmacy, Deraya University, Minia, EGY; 8 Faculty of Medicine, Minia University, Minia, EGY

**Keywords:** total knee replacement (tkr), network meta-analysis, hybrid, uncemented, cemented tka

## Abstract

In this study, we aim to explore the differences among the three types of fixation methods for the components of the knee joint in patients who underwent total knee arthroplasty (TKA). These methods are cemented, uncemented, and hybrid fixation. Cemented fixation means that a special type of grout is used to attach the components to the bone. Uncemented fixation means that the components are designed to fit tightly into the bone and allow new bone growth to secure them. Hybrid fixation means that a combination of cemented and uncemented fixation is used for different components. We searched four online databases to find studies relevant to our research question. We use the R program (R Foundation for Statistical Computing, Vienna, Austria) for network meta-analysis (NMA) to analyze the data from the studies. We calculate the mean difference (MD) and the 95% confidence interval (CI) for each outcome, which are statistical measures of the difference and the uncertainty between methods. We use these measures for continuous outcomes, meaning they can have any value. For dichotomous outcomes, meaning they can only have two values, we use the risk ratio (RR) and the 95% CI, which are statistical measures of the relative risk and the uncertainty between methods. We assess the quality of randomized controlled trials, which are studies that randomly assign participants to different methods, using the Cochrane Risk of Bias Assessment Tool 1, a tool that evaluates the potential biases in the studies. We include 21 studies, and our analysis shows that cemented TKA reveals a statistically significant decrease in pain with hybrid TKA (MD = -2.82). That said, we find no significant differences between uncemented and cemented or hybrid (MD = -0.80 and -2.02, respectively). The results show that there is no significant difference between uncemented TKA and cemented TKA or hybrid technique (RR = 0.87, 95% CI 0.35-2.14; RR = 0.73, 95% CI 0.22-2.39, respectively). Also, we find no significant difference between cemented TKA and hybrid TKA (RR = 0.84, 95% CI 0.24-2.93). Cemented TKA is associated with a lower risk of deep vein thrombosis (DVT) incidence rate. Moreover, it shows a significant decrease in pain compared to hybrid TKA. Future research is needed to compare among the three interventions.

## Introduction and background

Knee osteoarthritis is a common condition that can significantly affect the quality of life of a patient [1]. For people with severe knee damage, total knee arthroplasty (TKA) has been the preferred treatment option for the past 30 years. The first successful TKA was done in 1976 using a complete condylar prosthesis [2]. When nonoperative treatments have failed to improve a patient’s knee condition, TKA is one of the most effective surgical operations. Although the method and implants have improved, many revision TKAs are still performed every year. To reduce implant loosening failures, TKA fixation has been studied and refined throughout time. In the United States alone, tens of thousands of people undergo complete knee replacement surgery each year. The number reached well over 600,000 in 2010, and it is expected to rise considerably despite economic uncertainty [3]. Different surgical techniques are available to fix knee components, either cemented, uncemented, or hybrid [4,5].

Excellent long-term outcomes have been documented for the majority of patients who have had cemented tibial fixation. Aseptic loosening is a typical failure mechanism, especially in younger patients, despite cemented fixation being the gold standard for TKA. Because of the enhanced long-term life of the prosthesis [6], uncemented fixation continues to attract the attention of physicians who wish to utilize it in younger [7], more active patients to conserve bone stock, facilitate revision, and prevent issues associated with cementing [8].

Some researchers find that both cemented and uncemented TKAs have comparable functional outcomes and survival rates. Both surgical procedures have excellent success rates, and cemented TKA provides no further advantage. Uncemented fixation is preferable because it allows for stable fixation and shortens the duration of the surgery [9].

Debate is ongoing over whether the type of prosthetic fixation is optimum. Questions regarding cement fixation’s long-term durability and third-body wear have prompted interest in uncemented alternatives. This study aims to further understand the differences among cemented, uncemented, and hybrid fixation of knee components in TKA patients.

## Review

Methodology

We performed a network meta-analysis (NMA). We followed Preferred Reporting Items for Systematic Reviews and Meta-Analyses (PRISMA) guidelines, formulating our steps according to the Cochrane Handbook of Systematic Intervention [10,11].

Literature Search and Data Collection

We conducted our study using four electronic databases, PubMed, Scopus, Web of Science, and Cochrane, in April 2023. We used the following terms: ("Total Knee Replacement*" OR "Total Knee Arthroplast*" OR "total knee arthroscop*" OR "Total knee repair" OR "Total knee dislocation" OR "Total knee procedure" OR "Total knee operation" OR "Total knee surgery" OR "TKR" OR "TKA") AND ("Uncemented" OR "uncement" OR "Cementless" OR "Non-cemented" OR "Cemented OR cement*" OR "Hybrid").

We searched for any published results of ongoing studies on ongoing trials. Duplications were removed using Endnote (Clarivate Analytics, Philadelphia, PA) software, and all search received criteria assessed eligibility through a title, abstract, and full-text screening. We included the papers that matched our criteria in our study. We screened references of included studies manually for other related documents.

Eligibility Criteria and Study Selection

We performed our study following the guidelines outlined in the Cochrane Intervention Handbook guidelines. The screening was conducted using Microsoft Excel (2021 Edition, Microsoft Corp., Redmond, WA) in two steps: title and abstract and then full-text screening. Two independent researchers revised the references using previously established eligibility criteria. Our eligibility criteria encompassed (1) patients undergoing TKA; (2) intervention options including cemented, uncemented, or hybrid techniques; (3) comparators involving control, cemented, uncemented, or hybrid techniques; and (4) outcomes encompassing any evaluated outcome.

Methodological Quality Assessment

We implemented the methodology of our study using version 1 of Cochrane’s Risk of Bias (ROB) for randomized trials.

Data Extraction

Utilizing an Excel sheet, we obtained the following information: 1) summary and baseline characteristics, including study ID, TKA techniques, study design, site, trial registration, follow-up duration, age, percentage of the male population, VAS score, inclusion criteria, main outcomes, and conclusion; 2) study outcomes described as follows. Data extraction was carried out independently by two authors, with a third author consulted to resolve any conflicts.

Data Outcomes

The data outcomes include the change in Knee Society Score (KSS), change in maximum total point motion (MTPM) measured in millimeters, change in visual analog scale (VAS) score, rate of infections, and the incidence rate of DVT.

Data Synthesis

We used the R program (R Foundation for Statistical Computing, Vienna, Austria) for NMA to analyze data from studies. We calculated the mean difference (MD) and the 95% confidence interval (CI) for each outcome, which are statistical measures of the difference and the uncertainty between the methods. We used these measures for continuous outcomes, meaning they could have any value. For dichotomous outcomes, meaning they could only have two values, we used the risk ratio (RR) and the 95% CI, which are statistical measures of the relative risk and the uncertainty between methods. We tested the heterogeneity of data using the Chi-square test and I2 statistic, which are statistical tests of how consistent the results were across the studies. We used the random effect model, which is a statistical model that accounts for heterogeneity when the results are inconsistent across the studies. We considered the results to be inconsistent if *P*-value < 0.1 and I2 > 50, respectively.

Results

Literature Search

**Figure 1:**
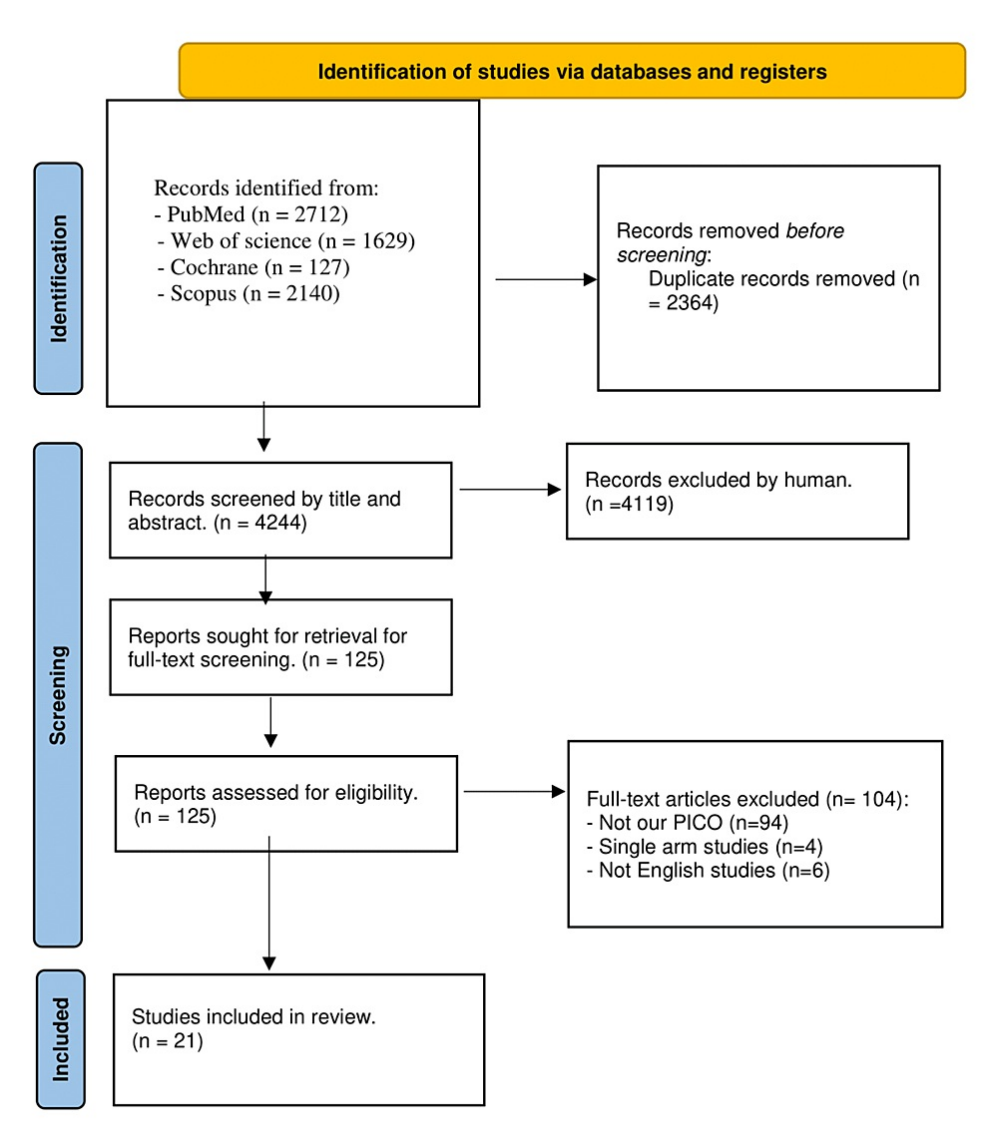
PRISMA flow diagram. PRISMA, Preferred Reporting Items for Systematic Reviews and Meta-Analyses; PICO, Participants, Intervention, Comparator, and Outcomes

We had 6,608 results after searching databases, and 4,244 results were obtained after the removal of duplicates. We screened the title and abstract, and 125 records were assessed by full-text screening. Finally, we obtained 21 [5,12-31] records for our eligibility criteria (Figure 1).

Quality Assessment

**Figure 2:**
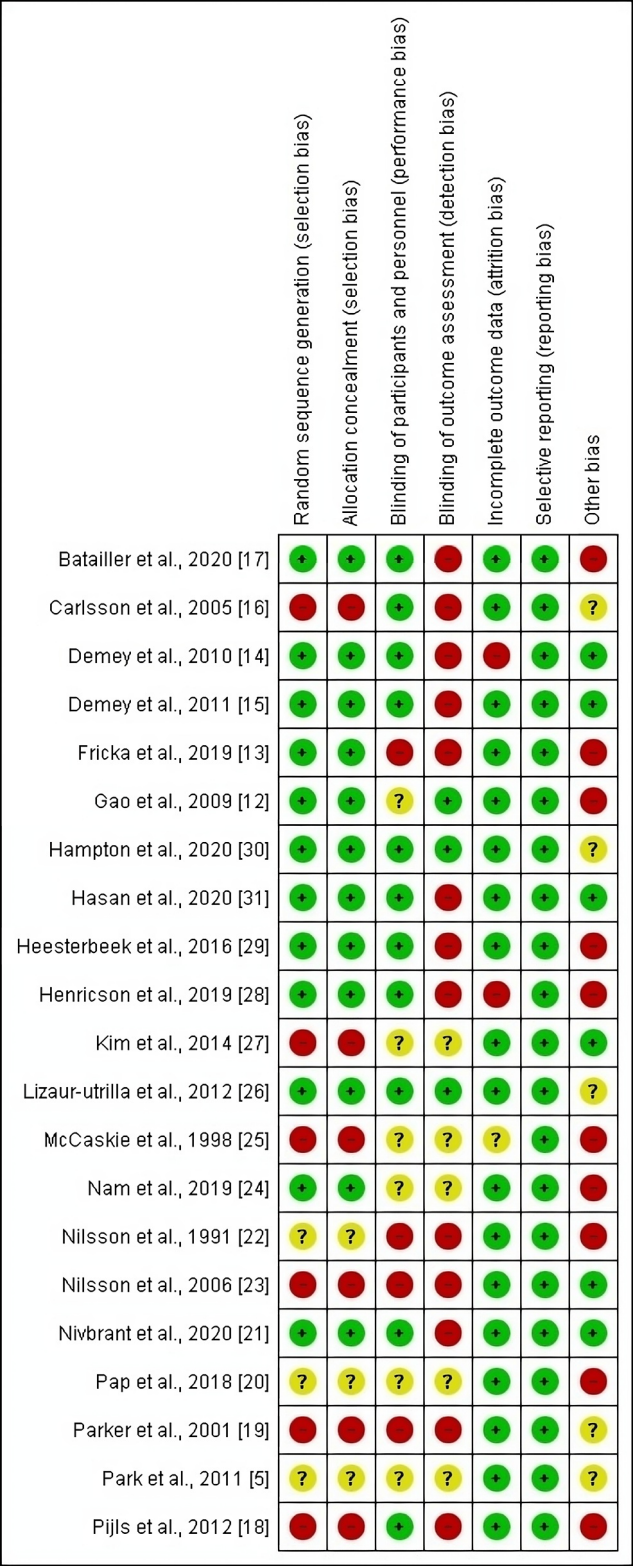
Risk-of-bias graph summary for RCTs. RCT, randomized controlled trial

Our included randomized controlled trials (RCTs) had a moderate ROB assessment, and the specific items and details are shown in Figure 2.

Summary and Baseline Characteristics of Included Studies

The total number of participants was 3,912, with a mean age of 50.5 years. Detailed information is shown in the summary and baseline table (Table 1).

**Table 1:** Summary and baseline characteristics of included studies. M, mean; SD, standard deviation; TKA, total knee arthroplasty; WOMAC, Western Ontario and McMaster University Osteoarthritis Index

Study ID	TKA techniques, *n *(%)	Study design (Registry)	Site	Follow-up (years)	Age (M ± SD) years	Male, *n* (%)	Implant side, *n* (%)	Underlying diagnosis, *n* (%)	Preoperative knee score (M ± SD)	VAS score (M ± SD)	Inclusion criteria	Main outcomes	Conclusions
Batailler et al. (2020) [17]	Cemented, 65 (50)	RCT (NCT00132587)	France	Mean (13)	72 ± 8.5	12 (18.5)	Right, 33 (50.8)	1. Osteoarthritis (OA), 56 (94.9) 2. Chondrocalcinosis, 3 (5.1)	48.9 ± 15.9	NR	1. Consecutive patients 2. Between 2004 and 2005 3. Age between 50 and 90 years 4. Between 18 and 50 years 5. With an indication for primary TKA	1. International Knee Society Score (KSS) 2. Change in the hip knee ankle angle	At a minimum follow-up of 10 years, there were no significant differences between cemented TKA and hybrid TKA for the survivorship, the complication rate, the clinical scores, or the radiological signs of loosening.
	Hybrid, 65 (50)				72.5 ± 7.3	20 (30.8)	Right, 36 (55.4)	1. OA, 58 (98.3) 2. Chondrocalcinosis, 1 (1.7)	49.3 ± 17.8				
Carlsson et al. (2005) [16]	Cemented, 29 (33.72)	Not registered (NR)	Sweden	Mean (2)	72 ± 7	7 (24.138)	1. Right, 14 (48.27) 2. Left, 15 (51.72)	OA	41 ± 12.75	NR	1. From 1992 to 1995 2. Patients with knee OA 3. Aged 40 (SD 11) years 4. TKRs were stratified into one of the two series	1. Radio Stereometry Assessment (RSA) outcomes 2. International KSS	Cementing of the tibial component offers more stable bone-implant contact for five years compared to uncemented fixation. When using uncemented components, however, there is evidence that augmenting a porous surface with hydroxyapatite may mean less motion between implant and bone after the initial postoperative year.
	Uncemented, 27 (31.4)				74 ± 6	7 (25.93)	1. Right, 17 (62.69) 2. Left, 10 (37.04)		40 ± 11				
	Uncemented hydroxyapatite, 30 (34.88)				72 ± 5	8 (26.67)	1. Right, 11 (36.67) 2. Left, 19 (63.33)		48 ± 16				
Demey et al. (2010) [14]	Cemented, 54 (50.47)	RCT	France		71 ± 8.6	12 (22.22)	1. Right, 26 (48.15) 2. Left, 28 (51.85)	1. OA, 51 (94.44) 2. Chondrocalcinosis, 3 (5.56)	NR	NR	1. From 2004 to 2005 2. Consecutive unilateral primary TKA 3. A patient aged 50-90 years 4. Under-diagnosis of OA or chondrocalcinosis	1. Hemoglobin levels 2. Hematocrit levels 3. Postoperative drainage and calculated blood loss	Cementing the femoral component during TKA does not appear to influence the amount of perioperative blood loss or the need for postoperative blood transfusion.
	Hybrid, 53 (48.53)				72.8 ± 6.6	16 (30.19)	1. Right, 27 (50.94) 2. Left, 26 (49.06)	1. OA, 52 (98.11) 2. Chondrocalcinosis, 1 (1.89)					
Demey et al. (2011) [15]	Cemented, 65 (50)	RCT	France	2.67 (SD 0.5)	72 ± 8.5	12 (18.5)	Right, 33 (50.8)	1. OA, 56 (94.9) 2. Chondrocalcinosis, 3 (5.1)	48.9 ± 15.9	NR	1. Between 2004 and 2005 2. Consecutive primary TKA 3. A patient age of 50-90 years 4. Under-diagnosis of OA or chondrocalcinosis	1. Knee scores 2. Function scores 3. Flexion scores	Cementing the femoral component of TKA does not appear to influence the clinical results. A longer follow-up period is required to determine the influence of radiological findings on final outcomes.
	Hybrid, 65 (50)				72.5 ± 7.3	20 (30.8)	Right, 36 (55.4)	1. OA, 58 (98.3) 2. Chondrocalcinosis, 1 (1.7)	49.3 ± 17.8				
Fricka et al. (2019) [13]	Cemented, 44 (51.76)	RCT	UK	At least (5)	Mean (58.4)	13 (29.55)	NR	OA	NR	NR	1. From May 2010 to February 2012 2. Mean age (58.9) years 3. Consecutive TKA 4. Under-diagnosis of OA or chondrocalcinosis	1. KSS 2. Oxford knee scores 3. Visual analog scale (VAS) scores	Uncemented and cemented TKA had equivalent patient-reported outcomes and survivorship at midterm follow-up. Updates are planned at 10- and 15-year intervals to observe long-term modes of failure between these two methods of fixation.
	Uncemented, 41 (48.24)				Mean (59.8)	15 (36.59)							
Gao et al. (2009) [12]	Cemented	RCT	Sweden	Mean (2)	<60	NR	NR	OA or OA secondary to trauma	NR	NR	1. Age less than 60 years 2. TKA due to primary OA or OA secondary to trauma 3. A body weight of less than 120 kg	1. Knee scores 2. Maximum total point motion (MTPM) measurements	These findings suggest that an uncemented and non-HA-coated femoral component may behave equally as well as a cemented one in the long term.
	Uncemented												
Hampton et al. (2020) [30]	Cemented, 41 (53.25)	RCT	UK	Mean (13.2)	63 ± 3.5	22 (48.9)	Right, 21 (46.7)	OA	NR	NR	1. Patients under the age of 70 years 2. With symptomatic OA of the knee 3. Primary TKA indicated 4. Able to understand the study and willing to return for follow-up	1. Several revisions 2. Knee-specific Oxford score 3. KSS 4. The global Short Form 12 (SF-12) scores	Use of an uncemented trabecular metal tibial implant can afford better long-term clinical outcomes when compared to cemented tibial components of a matched design. However, both have excellent survivorship up to 15 years after implantation.
	TMT, 36 (46.75)				64 ± 5	25 (55.6)	Right, 26 (57.8)						
Hasan et al. (2020) [31]	Cemented, 34 (49.28)	RCT (NCT02578446)	Sweden	Between 1 and 2	66 ± 6.3	18 (53)	Right, 15 (44)	OA	NR	NR	1. Between October 2015 and October 2016 2. OA Ahlbäck 22 stages II to IV 3. Males or non-pregnant females aged between 40 and 75 years 4. Had given informed consent	1. MTPM at 12 and 24 months 2. KSS 3. Knee Injury and Osteoarthritis Outcome Score (KOOS) over time 4. KOOS-quality of life over time	The uncemented TKA migrated more than the cemented TKA in the first two-year period. This difference was mainly due to a higher initial migration of the uncemented TKA in the first three postoperative months after which stabilization was observed in all but one mal-aligned and early revised TKA. Whether the biological fixation of the uncemented implants will result in increased long-term survivorship requires a longer follow-up.
	Uncemented, 35 (50.72)				65 ± 5.7	18 (51)	Right, 19 (54)						
Heesterbeek et al. (2016) [29]	Cemented, 16 (50)	RCT (NTR1315)	The Netherlands	At least 1	67 ± 6.5	6 (37.5)	1. Right, 10 (62.5) 2. Left, 6 (37.5)	NR	52.25 ± 12.25	66.25 ± 15.75	1. From January 2008 to April 2010 2. Adults on the waiting list for a total system revision of a primary TKA indicated 3. Had to be in stable health and free from or under treatment 4. Aged 65.5 (SD 6.2) years 5. Had given informed consent	1. KSS 2. Visual analog pain score (VAS) 3. KOOS score	At 24 months after revision, TKAs cemented and hybrid fixation replacements were equally stable. Unexpectedly, both groups had implants with >1 mm or >1 of micromotion although there were no clinical or radiographic signs of loosening. Whether these findings indicate the possibility of loosening with longer follow-up remains to be investigated.
	Hybrid, 16 (50)				64.5 ± 2.8	3 (18.75)	1. Right, 10 (62.5) 2. Left, 6 (37.5)		40 ± 15.5	54.5 ± 22.25			
Henricson et al. (2019) [28]	Cemented, 18 (52.94)	NR	Sweden	10	<60	18 (52.94)	NR	OA or post-traumatic OA	NR	NR	1. Between 2003 and 2004 2. Primary OA or post-traumatic OA 3. Age less than 60 years 4. Body weight less than 120 kg 5. Had given informed consent	1. MTPM overtime 2. KSS pain score 3. KSS function score	Uncemented fixation with titanium fiber mesh coating of the femoral component in TKA works equally as well as cemented fixation up to 10 years. Annual migration of 0.1 mm seems compatible with excellent long-term performance.
	Uncemented, 16 (47.06)												
Kim et al. (2014) [27]	Cemented, 80 (50)	RCT	South Korea	16.6 (SD 0.167)	54.3 ± 1	17 (21.25)	Bilateral, 80 (100)	OA	33.9 ± 7.67	NR	1. From January 1995 to March 1996 2. Patients ≤55 years 3. Bilateral, sequential, and simultaneous TKAs indicated	1. Total knee scores 2. Pain severity scores 3. Walking distance (%) 4. Range of motion	Long-term results of both uncemented and cemented TKAs were encouraging in patients with OA who were <55 years old. However, we found no evidence to prove the superiority of uncemented over cemented TKAs.
	Uncemented, 80 (50)								35.1 ± 5.67				
Lizaur-Utrilla et al. (2012) [26]	Cemented, 48 (51.61)	RCT	Spain	6.7 (SD 1.67)	52.0 ± 2.6	15 (31.25)	NR	Primary or post-traumatic OA	29.3 ± 8.1	NR	1. Patients who required primary TKA 2. Aged 55 years or younger 3. Diagnosis of primary or post-traumatic OA	1. WOMAC scores 2. Total knee scores 3. Range of motion 4. Several revisions	Uncemented TKA was found to be a reliable option in younger patients with OA. Although the revision rate and survival were similar in both groups, better clinical outcomes were obtained with uncemented tibial components.
	Uncemented, 45 (48.39)				51.4 ± 3.7	11 (24.44)			32.4 ± 6.7				
McCaskie et al. (1998) [25]	Cemented, 81 (58.27)	RCT	UK	Mean (5)	68.8 ± 8.2	32 (40)	NR	Primary or post-traumatic OA	NR	NR	1. Between June 1987 and December 1990 2. Aged 55 years or younger 3. Diagnosis of primary or post-traumatic OA 4. Had given informed consent	1. KSS 2. Nottingham Knee Score	At five years, our clinical results would not support the use of the more expensive uncemented fixation, whereas the radiological results are of unknown significance. Longer follow-up will determine any changes in the results and conclusions.
	Uncemented, 58 (41.73)				70.2 ± 7.2	26 (46)							
Nam et al. (2019) [24]	Cemented, 65 (46.1)	RCT (NCT03683992)	USA	2.075 (SD 0.275)	63 ± 7.6	31 (47.69)	NR	Arthritis	NR	NR	1. From February 2014 to November 2016 2. Age between 18 and 75 years 3. A primary TKA for a diagnosis of arthritis 4. The patient’s willingness to be randomized to be treated with a cemented or uncemented TKA	1. Estimated blood loss 2. Hemoglobin level 3. VAS score	This study demonstrated that a recently introduced uncemented TKA had results, both perioperatively and at an average of two years postoperatively that were equivalent to those of its cemented predecessor, without any aseptic failures of either implant. Thus, this study justifies continued surveillance of this device to elucidate both its survivorship and whether it can provide any long-term benefits.
	Uncemented, 76 (53.9)			2.1 (SD 0.325)	61.3 ± 7	40 (52.63)							
Nilsson et al. (1991) [22]	Cemented, 25 (58.14)	RCT	Sweden	Mean (2)	68.44 ± 5.81	5 (20)	NR	Rheumatoid arthritis and primary OA	45.54 ± 15.076	NR	1. Consecutive patients with OA or rheumatoid arthritis 2. Indicated for bi-tri compartmental knee arthroplasty 3. A primary TKA for a diagnosis of arthritis 4. Aged 68.44 (SD 5.81) years	1. MTPM measurements 2. Range of motion 3. Total knee score	There were no statistically significant differences between cemented and uncemented prostheses in either the OA or the RA group. The fixation in the RA patients did not significantly differ from that of the OA patients, perhaps because the RA patients had lower weight and were living a more sedentary life.
	Uncemented, 18 (41.86)				66.22 ± 9.72	3 (16.67)			46.67 ± 12.11				
Nilsson et al. (2006) [23]	Cemented, 34 (35.05)	RCT	Sweden	Mean (2)	55.5 ±7.5	14 (41)	NR	Rheumatoid arthritis and primary OA	28 ± 16	NR	1. Consecutive knees (85 patients) with OA or inflammatory arthritis having TKA 2. Having different types of fixation of the tibial component 3. Primary or secondary OA or inflammatory arthritis 4. Age younger than 65 years 5. A body weight of less than 150 kg	1. KSS 2. Knee Society Function Scores 3. Postoperative migration for three types of fixation	Uncemented fixation using hydroxy-apatite-coated implants without screws seems to be the best solution for the younger patient.
	Uncemented hydroxyapatite, 63 (64.95)				56.22 ± 7.277	24 (38.1)			23 ±14.88				
Nivbrant et al. (2020) [21]	Cemented, 51 (51)	RCT (ACTRN12619001024134)	Australia	Mean (2)	67.8 ± 8	NR	NR	OA	NR	6 ± 2.3	1. Symptomatic OA is eligible for a primary total knee replacement 2. Age from 45 to 90 years	1. MTPM measurements 2. Clinical improvement scores	Late ongoing subsidence and high maximum total point motion in our patients who underwent uncemented tibial fixation raise concerns about the fixation stability of ACS uncemented posterior stabilized knee replacements. Cemented tibial components were stable. Thus, we advise caution regarding the use of uncemented tibial components and recommend tibial fixation with cement for the ACS posterior stabilized total knee replacement.
	Uncemented, 49 (49)				68.8 ± 7.5					6 ± 2.3			
Pap et al. (2018) [20]	Cemented, 142 (51.08)	RCT	Hungary	Mean (2)	69 ± 14	78 (54.93)	NR	OA	48 ± 12	NR	1. Symptomatic OA is eligible for primary total knee replacement 2. Patients who underwent total knee replacement 3. Between September 2012 and February 2017 4. Mean age 69 (SD 14) years, 59 (SD 10) years, respectively 5. Written informed consent was obtained from each patient.	1. KSS 2. Pain severity score 3. Walking distance	We used the combination of titanium plasma spray and the hydroxyapatite porous coating technique in uncemented prostheses. Our results showed us that the novel Sanat Swing uncemented total knee prosthesis could be a great alternative to cemented TKA.
	Uncemented, 136 (48.92)				59 ± 10	83 (61.03)			47 ± 16				
Park and Kim (2011) [19]	Cemented, 50 (50)	RCT	South Korea	13.6 (SD 0.25)	58.4 ± 4	11 (22)	NR	Symptomatic OA	32.9 ± 12.75	NR	1. Patients (106 knees) who underwent sequential simultaneous bilateral TKR 2. Between January 1997 and February 1998 3. Between September 2012 and February 2017 4. Symptomatic OA 5. Written informed consent was obtained from each patient.	1. Total knee score 2. Mean function score 3. The walking distance	The rate of survival of the cemented tibial component was 100% and 98% for the uncemented tibial component. No osteolysis was identified in either group. Our data have shown no advantage of uncemented over cemented components in total knee replacement.
	Uncemented, 50 (50)								34.4 ± 10.75				
Parker et al. (2001) [5]	Uncemented, 52 (52)	RCT	Canada	Mean (12.8)	66.9 ± 6.2	33 (66)	NR	OA	NR	NR	1. Between January 1987 and December 1988 2. Patients with a diagnosis of OA 3. The average patient age was 66.7 years 4. Written informed consent was obtained from each patient.	Survival curve for all prostheses	With the elimination of poor design features related to the patellofemoral articulation and thin tibial poly-ethylene, cruciate-retaining total knee arthroplasties can yield good durable results, whether uncemented or hybrid fixation is used.
	Hybrid, 48 (48)				66.6 ± 6.35	21 (42)							
Pijls et al. (2012) [18]	Cemented, 24 (35.29)	RCT	The Netherlands	11-16	69 ± 8.6	6 (25)	NR	OA or rheumatoid arthritis	27 ± 11	NR	1. Consecutive posterior cruciate-retaining TKAs 2. Of OA or rheumatoid arthritis 3. Between 1993 and 1998 4. Written informed consent was obtained from each patient.	1. KSS function score 2. KSS score 3. Femorotibial angle	HA reduces migration of uncemented tibial components. This beneficial effect lasts for more than 10 years. Cemented components showed the lowest migration. Longitudinal follow-up of TKA with RSA allows early detection of secondary loosening.
	Uncemented, 20 (29.41)				65 ± 15	4 (20)			25 ± 21				
	Uncemented hydroxyapatite, 24 (35.29)				63 ± 11	3 (12.5)			22 ± 17				

Outcomes

**Figure 3:**
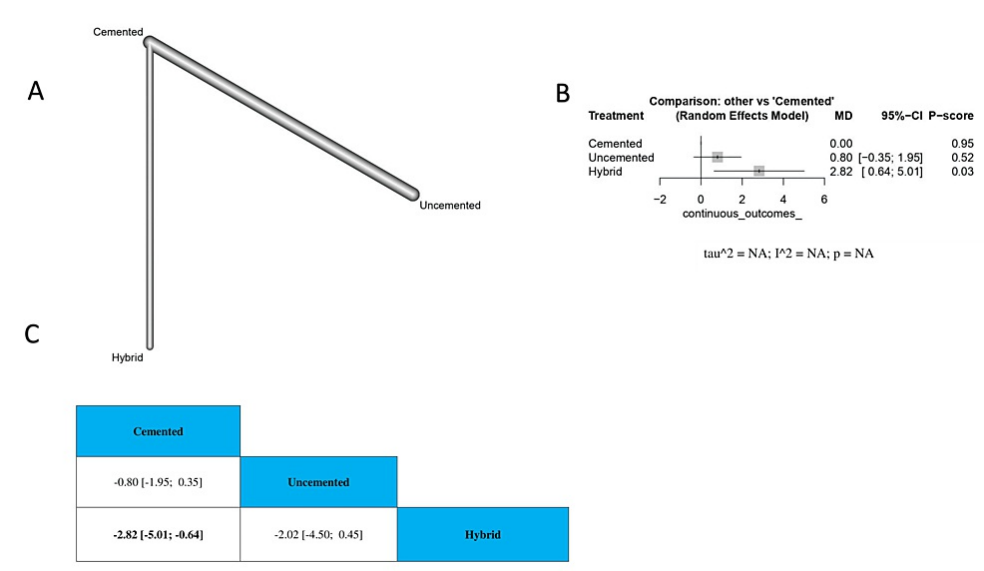
Change in pain (by VAS). (A) The network graph showing direct evidence between the evaluated interventions. (B) A forest plot comparing all interventions. (C) The league table represents the network meta-analysis estimates for all interventions' comparisons. VAS, visual analog scale; MD, mean difference

Change in pain (by VAS): Our analysis showed a statistically significant decrease in pain in the group that used cemented TKA compared to hybrid TKA (MD = -2.82, 95% CI -5.01 to -0.64). Also, we found no significant differences between these comparisons: (1) uncemented compared to cemented TKA, (2) uncemented compared to hybrid TKA (MD = -0.80, 95% CI -1.95 to 0.35; MD = -2.02, 95% CI -4.50 to 0.45, respectively) (Figure 3). The order of surgical techniques in terms of change in pain by VAS according to the *P*-score is cemented, uncemented, and hybrid (Figure 3).

Change in KSS at Two-Year Follow-Up

**Figure 4:**
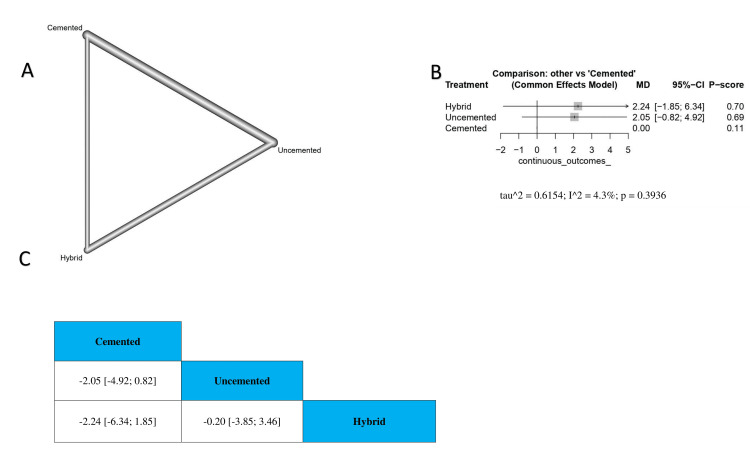
Change in KSS at the two-year follow-up. (A) A network graph showing direct evidence between the evaluated interventions. (B) A forest plot comparing all interventions. (C) The league table represents the network meta-analysis estimates for all interventions' comparisons. KSS, Knee Society Score; MD, mean difference; CI, confidence interval

Our analysis showed that cemented TKA revealed no significant difference in KSS with uncemented or hybrid TKA (MD = -2.05, 95% CI -4.92 to 0.82; MD = -2.24, 95% CI -6.34 to 1.85, respectively). Moreover, we found no significant differences between uncemented and hybrid TKA (MD = -0.20, 95% CI -3.85 to 3.46) (Figure 4). The order of surgical techniques in terms of change in KSS according to the *P*-score is hybrid, uncemented, and cemented (Figure 4).

Change in MTPM (mm) at Two-Year Follow-Up

**Figure 5:**
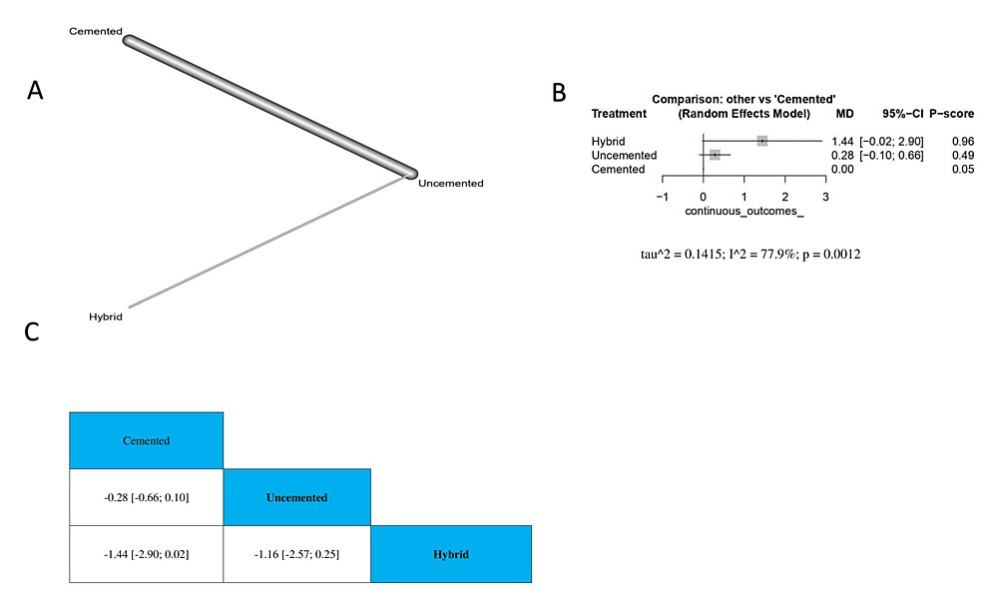
Change in MTPM (mm) at the two-year follow-up. (A) A network graph showing direct evidence between the evaluated interventions. (B) A forest plot comparing all interventions. (C) The league table represents the network meta-analysis estimates for all interventions' comparisons. MD, mean difference; CI, confidence interval; MTPM, maximum total point motion

Our analysis showed that cemented TKA revealed no significant difference in MTPM with uncemented or hybrid TKA (MD = -0.28, 95% CI -0.66 to 0.10; MD = -1.44, 95% CI -2.90 to 0.02, respectively). Moreover, we found no significant differences between uncemented and hybrid TKA (MD = -1.16, 95% CI -2.57 to 0.25) (Figure 5). The order of surgical techniques in terms of change in MTPM according to the *P*-score is hybrid, uncemented, and cemented (Figure 5).

The Incidence Rate of DVT

**Figure 6:**
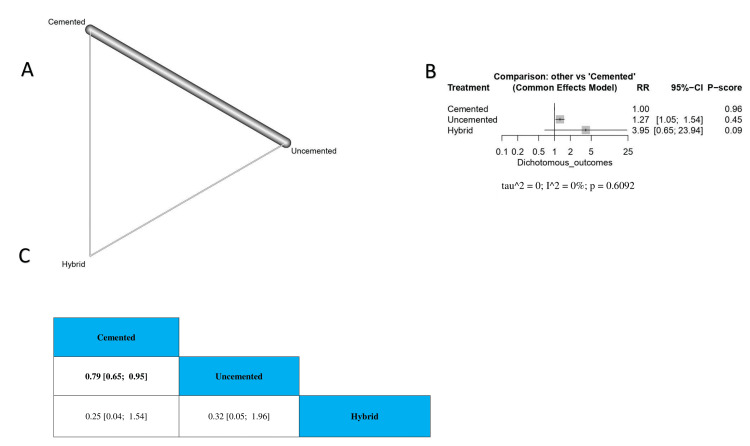
The incidence rate of DVT. (A) A network graph showing direct evidence between the evaluated interventions. (B) A forest plot comparing all interventions. (C) The league table represents the network meta-analysis estimates for all interventions' comparisons. RR, risk ratio; CI, confidence interval; DVT, deep vein thrombosis

The results showed that cemented TKA was associated with a lower risk of DVT incidence rate with uncemented TKA (RR = 0.79, 95% CI 0.65-0.95). Also, we found no significant difference between hybrid TKA and cemented or uncemented TKA (RR = 0.25, 95% CI 0.04-1.54; RR = 0.32, 95% CI 0.05-1.96, respectively) (Figure 6). The order of surgical techniques in terms of the incidence rate of DVT according to the *P*-score is cemented, uncemented, and hybrid (Figure 6).

The Incidence Rate of Infections

**Figure 7:**
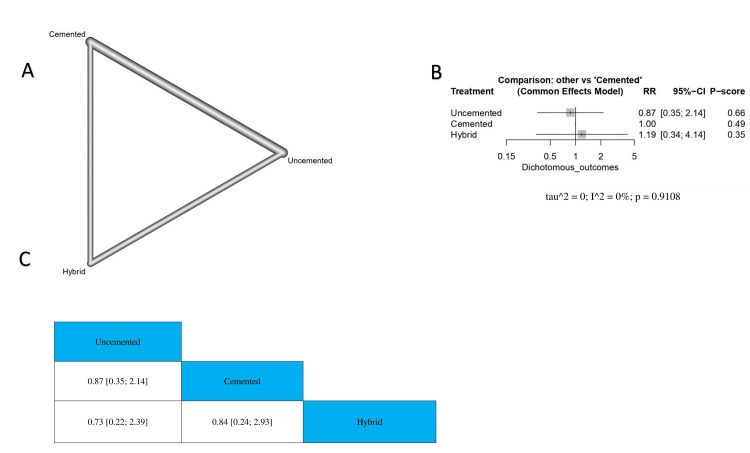
The incidence rate of infections. (A) Network graph showing direct evidence between the evaluated interventions. (B) A forest plot comparing all interventions. (C) The league table represents the network meta-analysis estimates for all interventions' comparisons. RR, risk ratio; CI, confidence interval

The results showed that there was no significant difference between uncemented TKA and cemented TKA or hybrid technique (RR = 0.87, 95% CI 0.35-2.14; RR = 0.73, 95% CI 0.22-2.39, respectively). Also, we found no significant difference between cemented TKA and hybrid TKA (RR = 0.84, 95% CI 0.24-2.93) (Figure 7). The order of surgical techniques in terms of the incidence rate of infections according to the *P*-score is uncemented, cemented, and hybrid (Figure 7).

Discussion

We found in our analysis that cemented TKA was superior to hybrid TKA in decreasing pain postoperatively. Several hypotheses could explain this finding, but they need to be further investigated by future studies. Therefore, more high-quality studies are needed to compare the efficacy and safety of uncemented and cemented TKA in different patient populations and follow-up periods. The factors that may influence the outcome of TKA, such as surgery duration, implant design, patient characteristics, and rehabilitation protocol, should also be considered and controlled. Also, cemented TKA was associated with a lower risk of DVT incidence rate compared to uncemented TKA. There are several possible explanations for the lower risk of DVT and pain in cemented TKA. One hypothesis is that cemented TKA provides better initial stability and less micromotion than hybrid or uncemented TKA, which reduces inflammation and thrombogenicity in the early postoperative period. Another hypothesis is that cemented TKA avoids the potential adverse effects of uncemented fixation, such as stress shielding, bone resorption, and delayed osseointegration, which could cause pain and instability in the long term. A third hypothesis is that cemented TKA allows for better alignment and balance of the knee joint, which enhances the biomechanical function and reduces the stress on the implant and surrounding tissues.

DVT can cause life-threatening consequences such as pulmonary embolism. Hence, it is essential to avoid and manage DVT after TKA with suitable prevention and monitoring. The type of fixation may not greatly affect the likelihood of DVT, but other aspects such as patient features, surgical method, implant shape, and post-surgery care may have an impact on the result. A previous systematic review and meta-analysis [32] comparing cemented and uncemented TKA showed no significant difference between cemented and uncemented TKA in the rate of DVT. The authors reported that 4.6% of patients in the uncemented group and 6.9% of patients in the cemented group developed DVT, with a relative risk of 0.81 (95% CI 0.47-1.39, *P* = 0.44). Another meta-analysis by Prasad et al. [33] showed no significant difference between cemented and uncemented TKA in the DVT outcome. This finding contradicts our results, which revealed that cemented TKA was associated with a lower incidence of DVT.

Pain is a usual way to assess the outcome of TKA and can impact the patient’s happiness and well-being. The type of fixation may not significantly affect the pain intensity, but other aspects such as implant shape, surgical method, patient features, and post-surgery care may have an effect. According to a systematic review and meta-analysis by Wang et al. [34], there was no significant difference between hybrid and cemented TKA in terms of pain by VAS score. They reported that the mean VAS score at the final follow-up was 0.84 in the hybrid group and 0.86 in the cemented group, with an MD of -0.02, 95% CI -0.11 to 0.07, and *P* = 0.64. This is different from our results, which revealed that cemented TKA showed a significant decrease in pain compared to hybrid TKA. To our knowledge, this is the first network meta-analysis constituting a comparison of the three techniques of TKA with a relatively large sample size. Our recommendations are further studies with good methods to establish and generalize the results of outcomes. We emphasize the need for more well-designed RCTs that directly compare the cemented, uncemented, and hybrid TKA to obtain generalizable results supported by a high level of evidence.

## Conclusions

We concluded that cemented TKA is associated with a lower risk of DVT and a lower pain level compared to hybrid TKA. However, the study did not find significant differences when comparing the outcomes of cemented, uncemented, and hybrid TKA with factors such as durability, infection rate, revision rate, and functional recovery. Therefore, we suggest that future research is needed to compare the three interventions in terms of these factors.
